# {2-Hydr­oxy-*N*′-[1-(2-oxidophenyl)ethyl­idene]benzohydrazidato}morpholinecopper(II)

**DOI:** 10.1107/S1600536809042810

**Published:** 2009-10-23

**Authors:** Song-Zhu Lin, Ruo-Kun Jia, Yan-Lin Yuan, Peng Zhan

**Affiliations:** a Chemical Engineering Institute, Northeast Dianli University, Jilin, Jilin 132012, People’s Republic of China

## Abstract

The Cu^II^ ion in the title complex, [Cu(C_15_H_12_N_2_O_3_)(C_4_H_9_NO)], is coordinated by one carbonyl O atom, one hydrazine N atom and one phenolate O atom from the doubly deprotonated tridentate ligand and one N atom from a morpholine mol­ecule, forming a distorted *trans*-CuN_2_O_2_ square-planar coordination geometry. An intra­molecular O—H⋯N hydrogen bond occurs within the ligand, generating an *S*(6) ring.

## Related literature

For background to aroylhydrazone derivatives, see: Singh (1992 ▶); Liu *et al.* (2003 ▶); Bai *et al.* (2005 ▶). For related structures, see: Gatto *et al.* (2004 ▶); Huo *et al.* (2004 ▶); Chen *et al.* (2009 ▶).
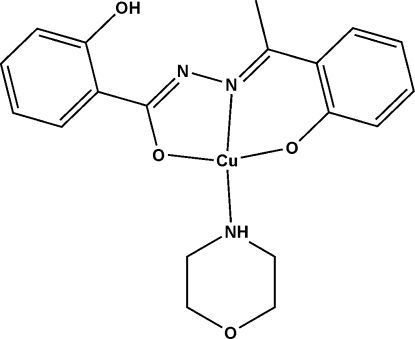


## Experimental

### 

#### Crystal data

[Cu(C_15_H_12_N_2_O_3_)(C_4_H_9_NO)]
*M* *_r_* = 418.93
Monoclinic, 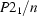

*a* = 9.220 (4) Å
*b* = 17.616 (9) Å
*c* = 12.023 (6) Å
β = 112.257 (14)°
*V* = 1807.4 (15) Å^3^
*Z* = 4
Mo *K*α radiation
μ = 1.24 mm^−1^
*T* = 293 K
0.26 × 0.17 × 0.14 mm

#### Data collection

Rigaku Weissenberg IP diffractometer
Absorption correction: multi-scan (*TEXRAY*; Molecular Structure Corporation, 1999 ▶) *T*_min_ = 0.769, *T*_max_ = 0.837
16528 measured reflections
4032 independent reflections
3273 reflections with *I* > 2σ(*I*)
*R*_int_ = 0.049

#### Refinement

*R*[*F* ^2^ > 2σ(*F* ^2^)] = 0.034
*wR*(*F* ^2^) = 0.089
*S* = 1.05
4032 reflections
246 parameters
H-atom parameters constrained
Δρ_max_ = 0.35 e Å^−3^
Δρ_min_ = −0.47 e Å^−3^

### 

Data collection: *TEXRAY* (Molecular Structure Corporation, 1999 ▶); cell refinement: *TEXRAY*; data reduction: *TEXSAN* (Mol­ecular Structure Corporation, 1999 ▶); program(s) used to solve structure: *SHELXS97* (Sheldrick, 2008 ▶); program(s) used to refine structure: *SHELXL97* (Sheldrick, 2008 ▶); molecular graphics: *ORTEP-3* (Farrugia, 1997 ▶); software used to prepare material for publication: *SHELXL97*.

## Supplementary Material

Crystal structure: contains datablocks I, global. DOI: 10.1107/S1600536809042810/hb5147sup1.cif

Structure factors: contains datablocks I. DOI: 10.1107/S1600536809042810/hb5147Isup2.hkl

Additional supplementary materials:  crystallographic information; 3D view; checkCIF report

## Figures and Tables

**Table 1:** Selected bond lengths (Å)

Cu1—O3	1.8702 (17)
Cu1—O2	1.9208 (16)
Cu1—N2	1.9409 (18)
Cu1—N3	2.0308 (19)

**Table 2:** Hydrogen-bond geometry (Å, °)

*D*—H⋯*A*	*D*—H	H⋯*A*	*D*⋯*A*	*D*—H⋯*A*
O1—H1*A*⋯N1	0.82	1.87	2.588 (3)	146

**Fig. 1.**
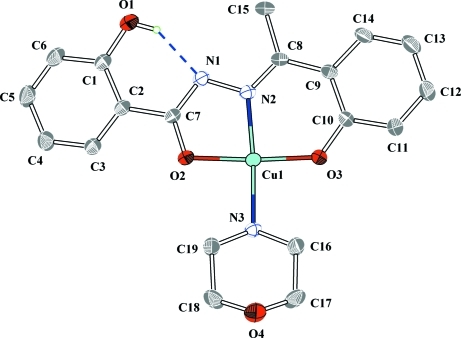
The molecular structure of (I), showing displacement ellipsoids at the 30% probability level for non-H atoms. Dashed lines indicate hydrogen bonding. Only H atoms involved in hydrogen bonds have been included.

## supplementary crystallographic information

### Comment

In the past decade, much attention has been focused on the study of aroylhydrazones derivative with aryl, aroyl and heteroaroyl Schiff bases due to their coordination abilities to metal ions (Singh *et al.*, 1992; Liu *et al.*, 2003; Bai *et al.*, 2005). Ongoing the study of aroylhydrazone complexes, we report here the synthesis and crystal structure of a new complex with 2-hydroxy-N'- (2-oxyphenyl-ethylidene)benzohydrazidate(2-) ligand (Fig. 1).

The title complex, (I), contains one copper(II) center having distorted quadrilateral coordination environment, one *O,N,O'*-tridentate ligand molecule and one coordinated morpholine molecule. There exists one intramolecular phenol-hydrazone O—H···N hydrogen bond in each ligand, forming a six-membered ring.

### Experimental

The ligand was prepared by the reaction of 2-hydroxyacetophenone and salicylhydrazine in a molar ratio of 1:1 under reflux in ethanol for 2 h. The white precipitate was collected, washed several times with ethanol and dried in *vacuo* (yield 79%). Morpholine (3 ml) was dropped into the mixture of 2-hydroxy-*N'*-(2-oxyphenyl- ethylidene)benzohydrazide (27 mg, 0.1 mmol) and Cu(Ac)_2_.2H_2_O (21 mg, 0.1 mmol) in methanol (10 ml). After stirring for 5 h, the reaction mixture was filtered and left to stand at room temperature. Green prisms of (I) were obtained by slow evaporation after 10 d. Analysis calculated for C_19_H_21_N_3_O_4_Cu: C 54.47, H 5.05, N 10.03%; found: C 53.99, H 5.01, N 10.29%.

### Refinement

H atoms bouded to phenolate O and morpholine N atoms were located in difference Fourier maps and were refined isotropically with O—H and N—H distance restraints of 0.82 and 0.91 Å, respectively. All other H atoms were placed in idealized positions and refined using a riding model [C—H = 0.93 Å and *U*_iso_(H) = 1.2*U*_eq_(C) for aromatic H atoms, C—H = 0.96 Å and *U*_iso_(H) = 1.5*U*_eq_(C) for methyl H atoms and C—H = 0.97 Å and *U*_iso_(H) = 1.2*U*_eq_(C) for the methylene H atoms].

### Crystal data

**Table:**

[Cu(C_15_H_12_N_2_O_3_)(C_4_H_9_NO)]	*F*(000) = 868
*M**_r_* = 418.93	*D*_x_ = 1.540 Mg m^−^^3^
Monoclinic, *P*2_1_/*n*	Mo *K*α radiation, λ = 0.71073 Å
Hall symbol: -P 2yn	Cell parameters from 4032 reflections
*a* = 9.220 (4) Å	θ = 3.3–27.5°
*b* = 17.616 (9) Å	µ = 1.24 mm^−^^1^
*c* = 12.023 (6) Å	*T* = 293 K
β = 112.257 (14)°	Prism, green
*V* = 1807.4 (15) Å^3^	0.26 × 0.17 × 0.14 mm
*Z* = 4	

### Data collection

**Table:**

Rigaku Weissenberg IP diffractometer	4032 independent reflections
Radiation source: fine-focus sealed tube	3273 reflections with *I* > 2σ(*I*)
graphite	*R*_int_ = 0.049
ω scans	θ_max_ = 27.5°, θ_min_ = 3.3°
Absorption correction: multi-scan (*TEXRAY*; Molecular Structure Corporation, 1999)	*h* = −11→10
*T*_min_ = 0.769, *T*_max_ = 0.837	*k* = −22→22
16528 measured reflections	*l* = −14→15

### Refinement

**Table:**

Refinement on *F*^2^	Primary atom site location: structure-invariant direct methods
Least-squares matrix: full	Secondary atom site location: difference Fourier map
*R*[*F*^2^ > 2σ(*F*^2^)] = 0.034	Hydrogen site location: inferred from neighbouring sites
*wR*(*F*^2^) = 0.089	H-atom parameters constrained
*S* = 1.05	*w* = 1/[σ^2^(*F*_o_^2^) + (0.042*P*)^2^ + 0.6843*P*] where *P* = (*F*_o_^2^ + 2*F*_c_^2^)/3
4032 reflections	(Δ/σ)_max_ = 0.001
246 parameters	Δρ_max_ = 0.35 e Å^−^^3^
0 restraints	Δρ_min_ = −0.47 e Å^−^^3^

### Special details

**Table:**

Geometry. All e.s.d.'s (except the e.s.d. in the dihedral angle between two l.s. planes) are estimated using the full covariance matrix. The cell e.s.d.'s are taken into account individually in the estimation of e.s.d.'s in distances, angles and torsion angles; correlations between e.s.d.'s in cell parameters are only used when they are defined by crystal symmetry. An approximate (isotropic) treatment of cell e.s.d.'s is used for estimating e.s.d.'s involving l.s. planes.
Refinement. Refinement of *F*^2^ against ALL reflections. The weighted *R*-factor *wR* and goodness of fit *S* are based on *F*^2^, conventional *R*-factors *R* are based on *F*, with *F* set to zero for negative *F*^2^. The threshold expression of *F*^2^ > σ(*F*^2^) is used only for calculating *R*-factors(gt) *etc*. and is not relevant to the choice of reflections for refinement. *R*-factors based on *F*^2^ are statistically about twice as large as those based on *F*, and *R*- factors based on ALL data will be even larger.

### Fractional atomic coordinates and isotropic or equivalent isotropic displacement parameters (Å^2^)

**Table:**

	*x*	*y*	*z*	*U*_iso_*/*U*_eq_	
Cu1	0.35584 (3)	0.512750 (15)	0.55888 (2)	0.03343 (10)	
O1	−0.2016 (2)	0.39749 (11)	0.30393 (17)	0.0635 (5)	
H1A	−0.1404	0.4330	0.3147	0.064 (9)*	
O2	0.24346 (16)	0.42406 (8)	0.57392 (13)	0.0392 (4)	
O3	0.46114 (18)	0.59329 (10)	0.52104 (14)	0.0479 (4)	
O4	0.7081 (2)	0.51533 (12)	0.97389 (16)	0.0626 (5)	
N1	0.06587 (19)	0.46639 (10)	0.39142 (17)	0.0373 (4)	
N2	0.17322 (19)	0.52585 (9)	0.41154 (16)	0.0334 (4)	
N3	0.5322 (2)	0.49568 (9)	0.72070 (16)	0.0343 (4)	
H3B	0.5980	0.4618	0.7068	0.042 (6)*	
C1	−0.1317 (3)	0.34097 (13)	0.3828 (2)	0.0438 (5)	
C2	0.0206 (2)	0.34834 (12)	0.4699 (2)	0.0369 (5)	
C3	0.0825 (3)	0.28792 (13)	0.5489 (2)	0.0428 (5)	
H3A	0.1829	0.2921	0.6075	0.051*	
C4	−0.0011 (3)	0.22263 (14)	0.5418 (3)	0.0534 (6)	
H4A	0.0428	0.1827	0.5946	0.064*	
C5	−0.1511 (3)	0.21633 (16)	0.4560 (3)	0.0618 (7)	
H5A	−0.2085	0.1722	0.4513	0.074*	
C6	−0.2160 (3)	0.27518 (16)	0.3775 (3)	0.0583 (7)	
H6A	−0.3174	0.2707	0.3205	0.070*	
C7	0.1169 (2)	0.41671 (12)	0.4806 (2)	0.0356 (5)	
C8	0.1354 (2)	0.58214 (12)	0.33475 (19)	0.0362 (5)	
C9	0.2478 (2)	0.64281 (12)	0.34607 (19)	0.0361 (5)	
C10	0.4016 (3)	0.64486 (12)	0.43607 (19)	0.0370 (5)	
C11	0.5022 (3)	0.70507 (13)	0.4351 (2)	0.0482 (6)	
H11A	0.6018	0.7072	0.4952	0.058*	
C12	0.4580 (3)	0.76027 (14)	0.3488 (3)	0.0570 (7)	
H12A	0.5275	0.7987	0.3494	0.068*	
C13	0.3082 (4)	0.75849 (15)	0.2602 (3)	0.0619 (7)	
H13A	0.2771	0.7958	0.2011	0.074*	
C14	0.2067 (3)	0.70213 (14)	0.2596 (2)	0.0503 (6)	
H14A	0.1065	0.7025	0.2002	0.060*	
C15	−0.0237 (3)	0.58277 (16)	0.2353 (2)	0.0536 (6)	
H15A	−0.0490	0.5326	0.2024	0.080*	
H15B	−0.0235	0.6172	0.1735	0.080*	
H15C	−0.1004	0.5989	0.2665	0.080*	
C16	0.6283 (3)	0.56298 (15)	0.7719 (2)	0.0539 (7)	
H16A	0.5626	0.6022	0.7849	0.065*	
H16B	0.6706	0.5824	0.7149	0.065*	
C17	0.7606 (3)	0.5460 (2)	0.8884 (2)	0.0656 (8)	
H17A	0.8319	0.5104	0.8740	0.079*	
H17B	0.8182	0.5924	0.9200	0.079*	
C18	0.6243 (4)	0.44769 (19)	0.9289 (2)	0.0773 (10)	
H18A	0.5881	0.4268	0.9885	0.093*	
H18B	0.6941	0.4108	0.9153	0.093*	
C19	0.4852 (3)	0.46037 (18)	0.8129 (2)	0.0630 (8)	
H19A	0.4348	0.4121	0.7832	0.076*	
H19B	0.4098	0.4928	0.8282	0.076*	

### Atomic displacement parameters (Å^2^)

**Table:**

	*U*^11^	*U*^22^	*U*^33^	*U*^12^	*U*^13^	*U*^23^
Cu1	0.02910 (14)	0.03649 (15)	0.02903 (15)	−0.00266 (10)	0.00461 (11)	0.00205 (10)
O1	0.0410 (9)	0.0587 (11)	0.0667 (12)	−0.0113 (8)	−0.0069 (9)	0.0105 (9)
O2	0.0315 (8)	0.0406 (8)	0.0368 (8)	−0.0046 (6)	0.0032 (7)	0.0024 (6)
O3	0.0385 (8)	0.0521 (10)	0.0427 (9)	−0.0085 (7)	0.0035 (7)	0.0171 (7)
O4	0.0669 (12)	0.0777 (13)	0.0314 (9)	−0.0159 (10)	0.0053 (9)	−0.0069 (8)
N1	0.0294 (9)	0.0389 (10)	0.0378 (10)	−0.0020 (7)	0.0060 (8)	−0.0014 (8)
N2	0.0295 (8)	0.0347 (10)	0.0316 (9)	0.0004 (7)	0.0067 (7)	−0.0024 (7)
N3	0.0321 (9)	0.0344 (9)	0.0314 (9)	0.0001 (7)	0.0065 (8)	−0.0045 (7)
C1	0.0394 (12)	0.0444 (13)	0.0434 (13)	−0.0061 (9)	0.0111 (10)	−0.0073 (10)
C2	0.0326 (10)	0.0384 (11)	0.0383 (11)	−0.0029 (8)	0.0118 (9)	−0.0091 (9)
C3	0.0402 (12)	0.0424 (12)	0.0438 (13)	−0.0020 (9)	0.0138 (11)	−0.0039 (10)
C4	0.0618 (16)	0.0403 (13)	0.0558 (15)	−0.0078 (11)	0.0197 (13)	−0.0032 (11)
C5	0.0701 (18)	0.0481 (15)	0.0636 (17)	−0.0251 (13)	0.0212 (15)	−0.0121 (13)
C6	0.0494 (15)	0.0606 (16)	0.0532 (15)	−0.0206 (12)	0.0063 (13)	−0.0138 (13)
C7	0.0297 (10)	0.0382 (11)	0.0377 (11)	0.0000 (8)	0.0115 (9)	−0.0047 (9)
C8	0.0335 (11)	0.0416 (12)	0.0304 (10)	0.0084 (8)	0.0087 (9)	−0.0003 (8)
C9	0.0391 (11)	0.0350 (11)	0.0341 (11)	0.0086 (8)	0.0139 (9)	0.0012 (8)
C10	0.0416 (11)	0.0356 (11)	0.0345 (11)	0.0025 (8)	0.0151 (10)	0.0017 (9)
C11	0.0489 (13)	0.0413 (13)	0.0524 (14)	−0.0024 (10)	0.0170 (12)	0.0038 (10)
C12	0.0651 (17)	0.0374 (13)	0.0692 (17)	−0.0018 (11)	0.0262 (15)	0.0089 (12)
C13	0.0736 (18)	0.0452 (15)	0.0617 (17)	0.0103 (13)	0.0196 (15)	0.0198 (13)
C14	0.0526 (14)	0.0436 (14)	0.0478 (14)	0.0109 (11)	0.0112 (12)	0.0126 (11)
C15	0.0411 (13)	0.0613 (16)	0.0451 (14)	0.0045 (11)	0.0014 (11)	0.0107 (11)
C16	0.0597 (15)	0.0538 (15)	0.0383 (12)	−0.0212 (12)	0.0072 (12)	−0.0059 (10)
C17	0.0515 (15)	0.092 (2)	0.0416 (14)	−0.0220 (15)	0.0043 (13)	−0.0141 (14)
C18	0.100 (2)	0.068 (2)	0.0369 (14)	−0.0222 (17)	−0.0044 (16)	0.0128 (13)
C19	0.0653 (17)	0.0730 (18)	0.0367 (13)	−0.0284 (14)	0.0036 (13)	0.0081 (12)

### Geometric parameters (Å, °)

**Table:**

Cu1—O3	1.8702 (17)	C6—H6A	0.9300
Cu1—O2	1.9208 (16)	C8—C9	1.459 (3)
Cu1—N2	1.9409 (18)	C8—C15	1.501 (3)
Cu1—N3	2.0308 (19)	C9—C14	1.421 (3)
O1—C1	1.357 (3)	C9—C10	1.421 (3)
O1—H1A	0.8200	C10—C11	1.412 (3)
O2—C7	1.283 (3)	C11—C12	1.366 (3)
O3—C10	1.321 (3)	C11—H11A	0.9300
O4—C17	1.399 (4)	C12—C13	1.389 (4)
O4—C18	1.412 (4)	C12—H12A	0.9300
N1—C7	1.325 (3)	C13—C14	1.362 (4)
N1—N2	1.398 (2)	C13—H13A	0.9300
N2—C8	1.309 (3)	C14—H14A	0.9300
N3—C16	1.468 (3)	C15—H15A	0.9600
N3—C19	1.472 (3)	C15—H15B	0.9600
N3—H3B	0.9100	C15—H15C	0.9600
C1—C6	1.384 (3)	C16—C17	1.499 (4)
C1—C2	1.404 (3)	C16—H16A	0.9700
C2—C3	1.397 (3)	C16—H16B	0.9700
C2—C7	1.473 (3)	C17—H17A	0.9700
C3—C4	1.370 (3)	C17—H17B	0.9700
C3—H3A	0.9300	C18—C19	1.512 (4)
C4—C5	1.381 (4)	C18—H18A	0.9700
C4—H4A	0.9300	C18—H18B	0.9700
C5—C6	1.377 (4)	C19—H19A	0.9700
C5—H5A	0.9300	C19—H19B	0.9700
			
O3—Cu1—O2	171.23 (7)	C10—C9—C8	123.93 (19)
O3—Cu1—N2	92.21 (7)	O3—C10—C11	116.2 (2)
O2—Cu1—N2	82.54 (7)	O3—C10—C9	125.0 (2)
O3—Cu1—N3	92.53 (7)	C11—C10—C9	118.8 (2)
O2—Cu1—N3	93.11 (7)	C12—C11—C10	122.2 (2)
N2—Cu1—N3	174.38 (8)	C12—C11—H11A	118.9
C1—O1—H1A	109.5	C10—C11—H11A	118.9
C7—O2—Cu1	110.14 (14)	C11—C12—C13	119.4 (2)
C10—O3—Cu1	127.42 (14)	C11—C12—H12A	120.3
C17—O4—C18	109.6 (2)	C13—C12—H12A	120.3
C7—N1—N2	110.10 (17)	C14—C13—C12	120.1 (2)
C8—N2—N1	117.62 (17)	C14—C13—H13A	119.9
C8—N2—Cu1	129.87 (15)	C12—C13—H13A	119.9
N1—N2—Cu1	112.43 (13)	C13—C14—C9	122.7 (2)
C16—N3—C19	109.2 (2)	C13—C14—H14A	118.7
C16—N3—Cu1	114.77 (15)	C9—C14—H14A	118.7
C19—N3—Cu1	115.24 (15)	C8—C15—H15A	109.5
C16—N3—H3B	105.6	C8—C15—H15B	109.5
C19—N3—H3B	105.6	H15A—C15—H15B	109.5
Cu1—N3—H3B	105.6	C8—C15—H15C	109.5
O1—C1—C6	118.2 (2)	H15A—C15—H15C	109.5
O1—C1—C2	122.0 (2)	H15B—C15—H15C	109.5
C6—C1—C2	119.8 (2)	N3—C16—C17	112.2 (2)
C3—C2—C1	118.2 (2)	N3—C16—H16A	109.2
C3—C2—C7	119.06 (19)	C17—C16—H16A	109.2
C1—C2—C7	122.7 (2)	N3—C16—H16B	109.2
C4—C3—C2	121.5 (2)	C17—C16—H16B	109.2
C4—C3—H3A	119.3	H16A—C16—H16B	107.9
C2—C3—H3A	119.3	O4—C17—C16	112.2 (2)
C3—C4—C5	119.7 (3)	O4—C17—H17A	109.2
C3—C4—H4A	120.2	C16—C17—H17A	109.2
C5—C4—H4A	120.2	O4—C17—H17B	109.2
C6—C5—C4	120.2 (2)	C16—C17—H17B	109.2
C6—C5—H5A	119.9	H17A—C17—H17B	107.9
C4—C5—H5A	119.9	O4—C18—C19	112.3 (2)
C5—C6—C1	120.6 (2)	O4—C18—H18A	109.2
C5—C6—H6A	119.7	C19—C18—H18A	109.2
C1—C6—H6A	119.7	O4—C18—H18B	109.2
O2—C7—N1	124.6 (2)	C19—C18—H18B	109.2
O2—C7—C2	118.6 (2)	H18A—C18—H18B	107.9
N1—C7—C2	116.84 (19)	N3—C19—C18	111.6 (2)
N2—C8—C9	119.94 (18)	N3—C19—H19A	109.3
N2—C8—C15	119.1 (2)	C18—C19—H19A	109.3
C9—C8—C15	121.0 (2)	N3—C19—H19B	109.3
C14—C9—C10	116.8 (2)	C18—C19—H19B	109.3
C14—C9—C8	119.2 (2)	H19A—C19—H19B	108.0
			
O3—Cu1—O2—C7	−49.8 (5)	N2—N1—C7—C2	−176.95 (18)
N2—Cu1—O2—C7	3.75 (14)	C3—C2—C7—O2	−11.5 (3)
N3—Cu1—O2—C7	−179.83 (14)	C1—C2—C7—O2	168.8 (2)
O2—Cu1—O3—C10	65.8 (5)	C3—C2—C7—N1	168.0 (2)
N2—Cu1—O3—C10	12.8 (2)	C1—C2—C7—N1	−11.7 (3)
N3—Cu1—O3—C10	−164.2 (2)	N1—N2—C8—C9	−175.30 (18)
C7—N1—N2—C8	−176.03 (19)	Cu1—N2—C8—C9	8.3 (3)
C7—N1—N2—Cu1	1.0 (2)	N1—N2—C8—C15	4.2 (3)
O3—Cu1—N2—C8	−13.1 (2)	Cu1—N2—C8—C15	−172.20 (17)
O2—Cu1—N2—C8	173.9 (2)	N2—C8—C9—C14	177.9 (2)
N3—Cu1—N2—C8	134.4 (7)	C15—C8—C9—C14	−1.6 (3)
O3—Cu1—N2—N1	170.31 (14)	N2—C8—C9—C10	1.1 (3)
O2—Cu1—N2—N1	−2.64 (14)	C15—C8—C9—C10	−178.4 (2)
N3—Cu1—N2—N1	−42.2 (8)	Cu1—O3—C10—C11	172.81 (17)
O3—Cu1—N3—C16	25.97 (19)	Cu1—O3—C10—C9	−8.7 (3)
O2—Cu1—N3—C16	−160.75 (18)	C14—C9—C10—O3	−177.8 (2)
N2—Cu1—N3—C16	−121.6 (7)	C8—C9—C10—O3	−0.9 (4)
O3—Cu1—N3—C19	154.11 (19)	C14—C9—C10—C11	0.6 (3)
O2—Cu1—N3—C19	−32.60 (19)	C8—C9—C10—C11	177.5 (2)
N2—Cu1—N3—C19	6.6 (8)	O3—C10—C11—C12	176.9 (2)
O1—C1—C2—C3	178.8 (2)	C9—C10—C11—C12	−1.7 (4)
C6—C1—C2—C3	0.3 (4)	C10—C11—C12—C13	1.4 (4)
O1—C1—C2—C7	−1.4 (4)	C11—C12—C13—C14	0.0 (4)
C6—C1—C2—C7	−180.0 (2)	C12—C13—C14—C9	−1.0 (4)
C1—C2—C3—C4	0.6 (4)	C10—C9—C14—C13	0.7 (4)
C7—C2—C3—C4	−179.1 (2)	C8—C9—C14—C13	−176.3 (3)
C2—C3—C4—C5	−0.9 (4)	C19—N3—C16—C17	51.4 (3)
C3—C4—C5—C6	0.3 (4)	Cu1—N3—C16—C17	−177.45 (19)
C4—C5—C6—C1	0.6 (5)	C18—O4—C17—C16	59.0 (4)
O1—C1—C6—C5	−179.5 (3)	N3—C16—C17—O4	−56.9 (3)
C2—C1—C6—C5	−0.9 (4)	C17—O4—C18—C19	−58.7 (4)
Cu1—O2—C7—N1	−4.7 (3)	C16—N3—C19—C18	−50.8 (3)
Cu1—O2—C7—C2	174.76 (15)	Cu1—N3—C19—C18	178.3 (2)
N2—N1—C7—O2	2.6 (3)	O4—C18—C19—N3	55.9 (4)

### Hydrogen-bond geometry (Å, °)

**Table:**

*D*—H···*A*	*D*—H	H···*A*	*D*···*A*	*D*—H···*A*
O1—H1A···N1	0.82	1.87	2.588 (3)	146
